# Improving stroke prevention therapy for patients with atrial fibrillation in primary care: protocol for a pragmatic, cluster-randomized trial

**DOI:** 10.1186/s13012-016-0523-2

**Published:** 2016-12-03

**Authors:** Theresa M. Lee, Noah M. Ivers, Sacha Bhatia, Debra A. Butt, Paul Dorian, Liisa Jaakkimainen, Kori Leblanc, Dan Legge, Dante Morra, Alissia Valentinis, Laura Wing, Jacqueline Young, Karen Tu

**Affiliations:** 1 Institute for Clinical Evaluation Sciences, 2075 Bayview Ave, Toronto, ON M4N 3M5 Canada; 2 Institute of Health Policy, Management and Evaluation, University of Toronto, 155 College Street, Suite 425, Toronto, ON M5T 3M6 Canada; 3 Department of Family and Community Medicine, Women’s College Hospital, 77 Grenville St, Toronto, ON M5S 1B3 Canada; 4 Department of Family and Community Medicine, University of Toronto, 500 University Avenue, 5th Floor, Toronto, ON M5G 1V7 Canada; 5 Women’s College Hospital Institute for Health System Solutions and Virtual Care, 76 Grenville St, Toronto, ON M5S 1B2 Canada; 6 Department of Family and Community Medicine, The Scarborough Hospital, 3030 Lawrence Avenue East, Suite 414, Scarborough, ON M1P 2V5 Canada; 7 Department of Medicine, University of Toronto, Suite RFE 3-805, 200 Elizabeth Street, Toronto, ON M5G 2C4 Canada; 8 Division of Cardiology, St. Michael’s Hospital, 30 Bond St, Toronto, ON M5B 1W8 Canada; 9 Sunnybrook Academic Family Health Team, 2075 Bayview Ave, Toronto, ON M4N 3M5 Canada; 10 Centre for Innovation in Complex Care, University Health Network, 200 Elizabeth Street Rm 13 N 1382, Toronto, ON M5G 2C4 Canada; 11 Leslie Dan Faculty of Pharmacy, University of Toronto, 144 College St, Toronto, ON M5S 3M2 Canada; 12 Institute for Better Health, Trillium Health Partners, 2200 Eglinton Avenue West, Mississauga, ON L5M 2N1 Canada; 13 Taddle Creek Family Health Team, 790 Bay St #522, Toronto, ON M5G 1N8 Canada; 14 Toronto Western Hospital Family Health Team, University Health Network, 399 Bathurst St, Toronto, ON M5T 2S8 Canada

**Keywords:** Atrial fibrillation, Stroke prevention, Multifaceted intervention, Cluster-randomized trial

## Abstract

**Background:**

The prevalence of atrial fibrillation (AF) is growing as the population ages, and at least 15% of ischemic strokes are attributed to AF. However, many high-risk AF patients are not offered guideline-recommended stroke prevention therapy due to a variety of system, provider, and patient-level barriers.

**Methods:**

We will conduct a pragmatic, cluster-randomized controlled trial randomizing primary care clinics to test a “toolkit” of quality improvement interventions in primary care. In keeping with the recommendations of the chronic care model to simultaneously activate patients and facilitate proactive care by providers, the toolkit includes provider-focused strategies (education, audit and feedback, electronic decision support, and reminders) plus patient-directed strategies (educational letters and reminders). The trial will include two feedback cycles at baseline and approximately 6 months and a final data collection at approximately 12 months. The study will be powered to show a difference of 10% in the primary outcome of proportion of patients receiving guideline-recommended stroke prevention therapy. Analysis will follow the intention-to-treat principle and will be blind to treatment allocation. Unit of analysis will be the patient; models will use generalized estimating equations to account for clustering at the clinical level.

**Discussion:**

Stroke prevention therapy using anticoagulation in patients with AF is known to reduce strokes by two thirds or more in clinical trials, but most studies indicate under-use of this treatment in real-world practice. If the toolkit successfully improves care for patients with AF, stakeholders will be engaged to facilitate broader application to maximize the potential to improve patient outcomes. The intervention toolkit tested in this project could also provide a model to improve quality of care for other chronic cardiovascular conditions managed in primary care.

**Trial registration:**

ClinicalTrials.gov (NCT01927445). Registered August 14, 2014 at https://clinicaltrials.gov/.

**Electronic supplementary material:**

The online version of this article (doi:10.1186/s13012-016-0523-2) contains supplementary material, which is available to authorized users.

## Background

Atrial fibrillation (AF) is a common and preventable cause of stroke [1]. The prevalence of AF is approximately 1% overall, but it accounts for 15% of all ischemic strokes and 33% of strokes in the elderly [2]. Such strokes result in permanent disability in 60% and death in 20% [3]. Aspirin reduces the relative risk of stroke in patients with AF by approximately 20–30% while anticoagulants reduce the relative risk of stroke in patients with AF by approximately 60–70% [4–7]. The traditional anticoagulation option, vitamin K antagonist (warfarin), may increase risk of bleeding [8, 9], has a narrow therapeutic index, and requires frequent blood tests (to monitor the international normalized ratio (INR) level) but remains an effective therapeutic option [8, 10]. Novel oral anticoagulants (NOACs) (e.g., dabigatran, rivaroxaban, apixaban) do not require blood monitoring as frequently and have been shown to have similar or superior efficacy to warfarin, lower rates of intracranial hemorrhage [11], and in some cases, reduced risk of bleeding [12]. The 2014 Canadian Cardiovascular Society (CCS) updated guidelines for atrial fibrillation emphasize that the vast majority of patients with AF would likely benefit from anticoagulation to reduce risk of stroke [13].

Despite the evidence that many AF-related strokes are preventable with proper therapy, the proportion of eligible patients receiving appropriate stroke prevention therapy remains far too low. A 2010 systematic review of 54 studies conducted around the world found that 50% of patients with AF at high risk of stroke did not receive anticoagulation [14]. A population-based study of patients over age 65 in Alberta published in 2011 found that only 49% of patients with a diagnosis of AF received anticoagulation, with no difference among those with highest and lowest risk of stroke [15]. A retrospective study in Ontario of hospitalizations for ischemic stroke between 2003 and 2007 showed that among a very high-risk group of patients with AF, a previous ischemic stroke or transient ischemic attack (TIA), and no known contraindications to anticoagulants, only 18% were receiving warfarin and in the desirable INR range on pre-admission [3]. Despite recent studies showing increase in use of oral anticoagulants (OAC) [16–18], anticoagulation therapy still remains suboptimal among patients with AF [19–22].

### Reasons for suboptimal care

Barriers to appropriate stroke prevention therapy may be present at the levels of the system, physician, and patient [23]. At the system level, specialized anticoagulation clinics may be associated with improved processes of care, [24] but are not available to most patients. A 2011 systematic review [25] concluded that a well-coordinated, structured approach is necessary for safe and effective management of anticoagulation, noting that this could occur in primary care [26]. Unfortunately, a Canadian survey of primary care clinics found inadequate coordination with laboratories, INR tracking systems, and use of reminders [27]. As a result, patients taking warfarin in Ontario are outside the therapeutic window up to 40% of the time [28]. Some primary care clinics have developed methods to monitor patients taking warfarin but rely on patients that are committed to regular blood tests. An ideal system of care would help primary care providers identify patients who neglect their blood tests, have discontinued their medications, or require a reassessment because the risk-benefit ratio of anticoagulants has changed due to development of new risk factors.

Even if such systems existed, physician’s knowledge and attitudes with respect to anticoagulants need to be addressed. The new CCS guidelines recommend that for most patients over age 65, the benefit of anticoagulants will outweigh the risks [13]. A meta-analysis of patient-level data from 8932 patients, covering 17.685 years of observation found that the benefit of anticoagulants increased with age, while the risks associated with aspirin increased with age and outweighed the benefits [29, 30]. However, physicians tend to substantially overestimate the risk of bleeding associated with anticoagulation, and to underestimate the benefits [31–33]. This may be because physicians tend to overestimate fall-risk in the elderly, [34] yet it is estimated that older patients taking warfarin must fall about 295 times per year for risks to outweigh benefits [35, 36]. It is important to accurately estimate the magnitude and severity of risk for both stroke and bleeding and to help patients weigh these risks [37]. This is particularly relevant in AF because AF-related stroke leads to permanent disability (physical and/or cognitive) in the majority of patients [38], and quality of life utility scores for stroke have been estimated to be worse than major bleeding [39]. In day-to-day clinical practice, identifying patients who may require changes to treatment, weighing risks against benefits, and communicating these risks to patients are time consuming and difficult [40].

A prospective, observational Canadian study found that patients with AF placed more value on the avoidance of stroke and less value on the avoidance of bleeding than their physicians [41]. In the context of under-use of formal risk assessment tools and anticoagulants, it is plausible that new tools supporting an evidence-informed, shared decision-making process with patients may lead to increased utilization of anticoagulants [42, 43]. The recent release of new guideline recommendations, the evidence that specific barriers are leading to suboptimal quality of care, and the presence of new medications emphasize the need for a comprehensive approach to knowledge translation in primary care to optimize patient management for this high-risk condition.

### Previous relevant trials aiming to improve stroke prevention therapy in AF

Three previous trials focused on patients using decision aids. A UK study that compared a computerized decision aid to printed guideline-based recommendations found decreased use of warfarin among low-risk patients but was unsuccessful in increasing its use among high-risk patients [44]. Two Canadian studies that compared patient decision aids to no intervention resulted in no change in the use of warfarin [36, 45]. One of these studies did observe an improvement in stroke prevention therapy at 3 months but no difference at 12 months [45], suggesting the need for longer-term strategies. A multifaceted approach that includes reminders for adherence might be more effective for long-term anticoagulation. Since risks change over time and because persistence with anticoagulation is known to decline significantly over time, longitudinal, multifaceted interventions may be more likely to improve uptake of stroke prevention therapy.

Two previous trials directed at providers have attempted to increase anticoagulation in patients with AF. One cluster-randomized trial in the USA compared audit and feedback against audit and feedback plus outreach visits and learning collaborative meetings, aiming to improve a variety of processes related to cardiovascular risk management [46]. There was no change in the rate of oral anticoagulation [46]. In another cluster-trial in the UK, investigators adapted guidelines and promoted them in educational meetings led by opinion leaders, as well as outreach visits [47]. There was a 10% increase in the proportion of patients with guideline-concordant stroke prevention, but the study was powered to find a 20% increase. Currently, two large cluster-trials are underway in Australia involving specialist support regarding specific cases and telephone-based educational outreach [48, 49]. Given the lack of studies conducted since NOACs have come to market, the results of these educational interventions will be of great interest. Yet, even if knowledge is addressed among providers and patients, sustained improvements in stroke prevention therapy cannot be achieved without addressing other barriers.

At the system level, “NHS Improvement” in the UK has identified AF as a priority topic, [50] encouraging appropriate stroke prevention therapy and providing tools to enable audit of anticoagulation use in patients at each risk level. In 2013, new quality outcomes framework benchmarks in the UK were implemented to encourage formal stroke risk assessment and use of anticoagulation [51]. Another approach was illustrated in a Dutch trial of a nurse-run, guideline-based specialty clinic for AF patients, which led to a statistically significant decrease in cardiovascular mortality compared to usual care (1.1% versus 3.9%), with no difference in major bleeding (1.7% in each arm) [52]. This supports the concept of interventions implementing standardized processes for patients with AF, but specialized clinics are not likely to be available to most patients with AF. There is a need for quality improvement initiatives that simultaneously addresses provider-level, patient-level, and organization-of-care-related barriers in primary care. The objective of this study is to test a “toolkit” of quality improvement interventions that aims to incorporate best evidence regarding its components and tailor intervention design to known determinants of stroke prevention treatment in primary care.

## Methods

### Study design

This is a pragmatic, cluster-randomized trial with two parallel arms and outcome analysis blinded to allocation. The cluster design was chosen to account for multi-physician clinics and to determine the effects at the patient level, provider levels. The protocol is registered at clinicaltrial.gov (NCT01927445). The Sunnybrook Health Sciences Centre research ethics office approved the study (075–2013). This trial will be conducted in conjunction with a similar study focused on chronic kidney disease (CKD) whereby the intervention group for this trial will serve as the control group for the CKD trial and vice versa (i.e., an “active-control” trial). Therefore, the randomization must take into account covariates related to CKD.

### Participants

Participants will be drawn from the primary care practices associated with the Electronic Medical Record Administrative data Linked Database (EMRALD) in Ontario, Canada. EMRALD is a unique data source that captures complete clinical patient charts already being used by family physicians (thereby avoiding the need for case report forms) [53, 54]. The electronic medical record (EMR) data are extracted and securely stored at the Institute for Clinical Evaluative Sciences (ICES), where it can be linked to population-level administrative health databases. EMRALD provides an opportunity for large-scale initiatives by facilitating centralized, automated chart abstraction, and data analysis using validated database algorithms to assess and provide feedback regarding quality of care. The platform has been used before to facilitate a quality improvement trial [55].

Eligible physicians are those participating in EMRALD, using PS Suite® EMR for at least 2 years, and have 100 or more rostered patients. These eligibility criteria are to ensure that physicians have had sufficient experience with their EMR and have adequate patient data to assess baseline and outcome measures. Eligible patients within EMRALD will have a physician diagnosis of AF recorded in their chart and >18 years of age. No distinction will be made between paroxysmal, persistent, and permanent AF. Analysis will be restricted to adult patients rostered to participating physicians since physicians may not start long-term medications for patients who are not regularly seen at their practice (thus excluding encounters such as “walk-in” patient visits).

At study commencement, 194 eligible physicians from 34 clinics with a total roster of 140,147 adult patients contributed data to EMRALD. These clinics are located across Ontario in urban, semi-urban, and rural settings. Comparisons conducted at ICES of the patient population of EMRALD physicians to the general primary care population in Ontario show that they are similar with respect to age and sex [56]. Participating physicians in EMRALD have access to the System for Audit and Feedback to Improve caRE (SAFIRE). SAFIRE is a secure, password-protected website used to provide performance feedback reports regarding quality of care for chronic disease. Data are available at both the aggregate and patient-specific levels. Prior to this trial, SAFIRE focused on comparing actual to guideline-recommended practice for patients with hypertension, diabetes, and/or ischemic heart disease. Physicians are able to compare their performance to other physicians that participate in EMRALD, and for physicians practicing in a group practice, physicians are able to compare their performance to other physicians within their own clinic, and their clinic performance compared to other clinics that participate in EMRALD.

### Allocation

To avoid contamination, all physicians (and patients) belonging to a practice with shared administrative resources will be randomly assigned in a cluster to the intervention or control arm. The allocation will be done centrally based on clinic (clusters) by an analyst to ensure concealment from the study investigators. Restricted randomization has been recommended for cluster trials when baseline data is available [57]. We will use minimization [58] to evenly distribute clinics across trial arms and to improve balance across the following baseline covariates using the free software “MINIM” [59]: the total number of primary care physicians in each clinic, number of patients over 65 years, average age of all patients, rural location, average years of experience, average years on the EMR, number of patients with hypertension, number of patients with ischemic heart disease, number of patients with diabetes, number of patients with atrial fibrillation, number of patients with stage 3+ CKD, number of patients meeting blood pressure target (140/90 or 130/80 if patient has diabetes), number of patients 65 and older with atrial fibrillation or have a CHADS2 ≥ 1 and an anticoagulation prescription, and number of patients between 50 and 80 years of age with stage 3+ CKD on a statin unless contraindicated. Using baseline data from the participating clinics, continuous variables will be classified as high or low using the median value as the cut-point. Unlike stratification, increasing the number of covariates in minimization does not lead to increased risk for imbalance [60]. The participating practices will be minimized simultaneously at the initiation of the trial, reducing risk of selection bias.

### Intervention

The control arm will receive “usual care” without any attempt to standardize treatment. Usual care clinics will be unaware of a study focusing on atrial fibrillation, and will continue to receive audit and feedback regarding their patients with other clinical conditions [55]. However, guidelines do not self-implement, and many trials of guideline implementation interventions are not successful in improving care [61]. This is in part explained by poor matching of interventions to barriers [62]. Thus, we have developed a multifaceted knowledge translation program to address many of the known barriers to optimal stroke prevention therapy (Table 1). A multidisciplinary team including family physicians, cardiologists, general internists, a pharmacist, graphic designers, and an EMR programmer provided input over the course of 2 years to refine the intervention components. During this time, we considered the literature regarding barriers to optimal stroke prevention therapy and how these could be addressed using feasible, evidence-based approaches. A pilot of the strategies in the intervention toolkit was conducted in a large primary care clinic where a co-investigator works as a family physician (AV) [17, 63]. We used continuous quality improvement methods, using rapid plan-do-study-act cycles to iteratively optimize the EMR tool format and design to ensure they met the needs of busy primary care clinicians. Evaluation of this pilot was mixed methods, including observed use of the tools and semi-structured interviews with 14 patients-provider dyads to assess usability of the EMR tools and preferences regarding implementation support [63].

**Table 1:** Matching barriers to intervention components

Examples of barriers to stroke prevention treatment	Strategies for quality improvement
Other patient issues may distract from AF management	EMR AF toolbar reminder
Provider awareness of new guidelines	Educational materials
Provider attitudes regarding bleeding risk with anticoagulants	Educational materials, risk calculator
Provider assessment and communication of risk/benefit	EMR risk tool added to each relevant patient chart with summary statements
Systems to monitor performance against standards and to identify patients without treatment	Audit and feedback with patient-level data
Systems to monitor patients on treatment	EMR AF treatment flow chart
Systems to identify patient non-adherence	EMR searches and reminders

The resulting multifaceted intervention will be presented to recipients in clinics of the intervention arm as a toolkit. A pragmatic approach will be taken in which providers and clinics will be welcome to use whichever aspects of the toolkit they choose. Below, we describe the evidence base for selection of the intervention components and the approach used to deliver them in this trial.

#### Printed educational materials for physicians

A Cochrane systematic review found that printed educational materials may have a beneficial effect on professional practices (median 4.3% increase in intended processes, interquartile range (IQR) −8.0 to 9.6%) [64]. Working with a multidisciplinary team of clinicians and designers, we have developed “AFib in One Page,” a summary of AF guideline recommendations relevant to primary care (http://afibreno.uhnopenlab.ca/wp-content/uploads/2014/10/afonepage.pdf). We will also append a one-page series of questions and answers specifically developed to provide brief evidence-based statements addressing the knowledge-related barriers described above (e.g., how to account for risk of bleeding due to risk of falling in the elderly) (http://afibreno.uhnopenlab.ca/wp-content/uploads/2014/11/faq.pdf). To validate the accuracy and utility of the educational material, input was sought from a multidisciplinary team including members of the CCS guideline committee.

#### Audit and feedback

A Cochrane systematic review found that audit and feedback increases healthcare professionals’ compliance with desired practice by 4.3% (IQR 0.5 to 16%) [57]. We have experienced conducting trials featuring audit and feedback to family physicians, having recently completed a 2-year trial in which participants received data regarding the proportion of patients with diabetes and/or heart disease meeting evidence-based targets [55]. Both aggregate and individual-level data will be provided using data from the physician’s own electronic patient charts through SAFIRE.

**Fig. 1:**
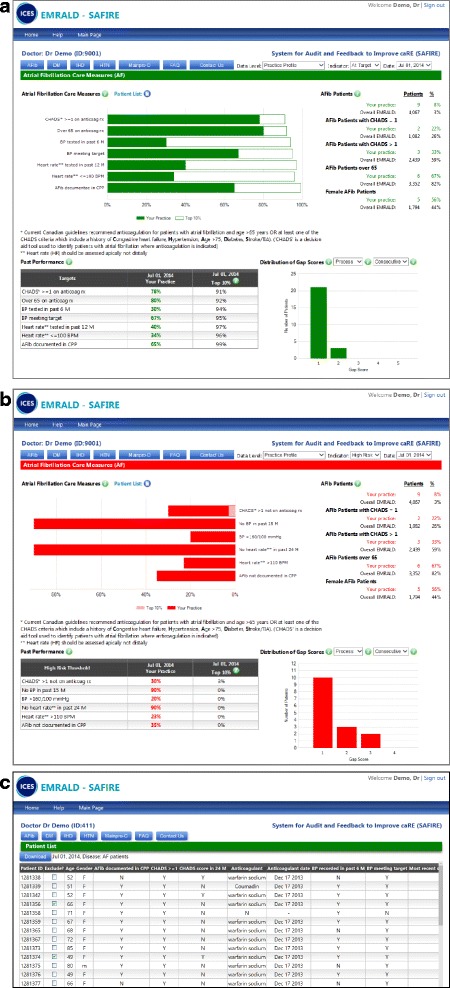
Examples of System for Audit and Feedback to Improve caRE reports. **a** System for Audit and Feedback to Improve caRE example of aggregate-level feedback report for atrial fibrillation at target. **b** System for Audit and Feedback to Improve caRE example of aggregate-level feedback report for atrial fibrillation: high risk. **c** Example of patient-specific feedback for atrial fibrillation

Performance will be compared against the median score of the top ten percent of peers, an evidence-based approach to provide an achievable benchmark of care [65]. In addition, drop-down menus allow the user to aggregate the data for all physicians in the practice and compare this to all other intervention practices to facilitate practice-level quality improvement strategies (see Figs. 1a, b for example feedback reports). The design of these reports will match those already delivered to all EMRALD participants (diabetes, heart disease, and hypertension) in addition to AF reports. Both raw numbers and tables are used to help users visualize achievements of quality indicators (and change over time). Based on a qualitative process evaluation of the aforementioned trial [66], the feedback presents both achievements of best-practice quality indicators, and of patients exceeding high-risk thresholds. The patient-level data can be used to sort by patients, any of the quality indicators, or high-risk thresholds. There is also an opportunity for users to exclude individual patients from future reports (see Fig. 1c).

Physicians (or their assigned delegate) can sign into a secure online platform to retrieve their data. New audits will be conducted every 6 months and feedback reports are typically available 1–3 months after data are collected. Continuing medical education (CME) credits are available to users who review the data and complete a worksheet that suggests possible action plans for each clinical condition (i.e., ask front office staff to arrange an appointment to discuss treatment options [see Additional file 1 for example worksheet]). EMRALD participants receive email notices when updated feedback is available. The emails will include instructions for delegates (usually a nominated administrative person in each clinic) to download and create a prioritized list of patients who may benefit from reassessment (e.g., those with CHADS2 > 1 and no anticoagulation or no recent blood pressure measurement).

#### EMR-based clinical decision support and reminders

**Fig. 2:**
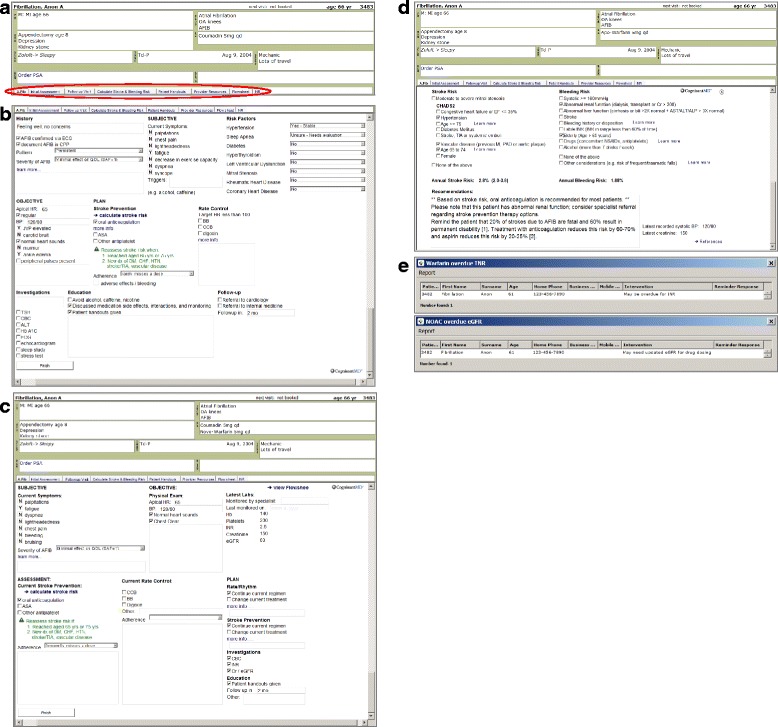
Screenshots of electronic medical record tools for atrial fibrillation. **a** Atrial fibrillation toolbar. **b** Structured template for atrial fibrillation initial assessment. **c** Structured template for atrial fibrillation follow-up visit. **d** Chart-based clinical decision aid for calculating stroke and bleeding risk. **e** Physician reminders for INR tracking and overdue renal function tests
- Templates for initial assessment or routine follow-up of patients with AF (Fig. 2b, c): Our pilot project provided strong impetus for the development of guided clinical encounter forms. Clinicians desired prompts regarding the essential items to cover when seeing a patient with AF and wanted to retain flexibility to respond to such prompts according to the competing priorities of a given encounter. With these principles in mind, we included in the toolkit EMR-based “custom forms” (i.e., templates) that can be inserted within the progress note. These follow a typical SOAP note format (i.e., subjective, objective, assessment, plan) familiar to family physicians and facilitate a brief assessment of both quality of life and adherence to stroke prevention treatment.
- AF-related stroke and bleeding risk calculator (Fig. 2d): Counseling regarding risks and benefits is challenging, even for patient/provider dyads with high numeracy. Primary care physicians often experience discordance when attempting to both integrate shared decision-making ideals and meet guideline recommendations [69]. This tool aids the provider to quickly calculate both risk of stroke with and without treatment and the risk of bleeding. The output will remind patients and providers that risk of stroke and risk of bleeding should not be weighed evenly, given that impact on quality-adjusted life-years (QALY) for bleeding is much smaller than for stroke.
- Reminders for INR tracking or overdue for renal function (Fig. 2e): Even if prescribed with warfarin, evidence suggests that patients may be in therapeutic range only about half the time [70]. Patients attending anticoagulation clinics with systematic, protocol-driven approaches to warfarin dosing spend more time in therapeutic range, while other physicians may have a tendency to underdose rather than overdose warfarin [71]. The goal of this tool is to provide physicians (or pharmacists associated with intervention clinics) with a system for tracking patients taking warfarin and assist providers in adjusting warfarin dose according to the INR result.

A recent systematic review of randomized trials found electronic clinical decision support systems increased the proportion of providers making appropriate prescriptions (odds ratio 1.57; 95% confidence interval (CI), 1.35 to 1.82). Although government-funded initiatives have been effective at increasing uptake of EMR systems in primary care in Ontario, there is a large division between simply using EMR as an electronic means of storing records and leveraging EMR data for enhancing provision of care. Crossing this division is challenging [67]; national surveys suggest that <15% of Canadian providers are leveraging EMR data to support quality improvement [68]. Therefore, we will provide simple-to-use EMR tools that can be easily activated within the charts of patients with AF at the point of care. Further information regarding coding and/or functionality of the tools below can be shared upon request:

- AF toolbar (Fig. 2a): The AF toolbar is a reminder system to prompt physicians to address AF when asymptomatic patients come to the office for other reasons. It will appear at the top of the chart of patients documented to have AF in the cumulative patient profile of the EMR record and will provide one-button access to the EMR-based AF tools described below. The toolbar includes quick-links to provider resources (e.g., medication dosing information) and patient handouts that use lay terms to describe AF, AF treatment, and management options (http://afibreno.uhnopenlab.ca/?page_id=155).

### Implementation support

A previous study examining a quality improvement intervention in Ontario found that even multidisciplinary primary care teams failed to take action upon receiving feedback reports indicating substandard performance on chronic disease management due to a lack of “performance management skill development” [72]. At the same time, high-cost interventions are unlikely to be scalable or replicated. Therefore, we aim to offer implementation support in a manner that could be sustainable, as described below.

With notification that the SAFIRE feedback is available, intervention clinics will be offered a virtual group presentation (i.e., a webinar) by research team members familiar with both the toolkit and the clinical aspects of AF. In advance of this meeting, we will attempt to identify local physician champions and key non-physician participants (i.e., data retrieval delegates) at each intervention clinic. The educational aspect of the presentation will review the guideline summary including approaches for risk assessment and information about NOACs and then introduce the functionality of the online feedback platform. Although the feedback is confidential for each physician, participants will be encouraged to discuss the results among their teams, providing an opportunity to problem solve and share best practices. The group presentation will also be used to introduce the EMR-based features of the toolkit. Research staff will then provide support to clinic administrators to install and activate the EMR-based tools. (The EMR-based reminders or tools can be “turned off” by the providers at any time.) Any participants not able to attend the group meeting will be provided with a video recording of the webinar and offered a personal phone meeting with similar objectives. One month after the initial presentation, each clinic will receive invitations for one-to-one assistance to troubleshoot any problems arising with the intervention components and to offer again help implementing the toolkit. With new feedback approximately 6 months later, research staff will contact the physicians via email to prompt them to review updated feedback reports, an infographic designed to encourage interest in improving the management of AF [Additional file 2], as well as to offer support for implementing or using the toolkit.

### Outcomes

The primary outcome is the proportion of patients with AF and an estimated yearly risk of stroke greater than 2% who are receiving stroke prevention therapy after one year. Specifically, the denominator will be patients aged ≥ 65 or who have a CHADS2 score ≥ 1. Offering such patients anticoagulation would be concordant with the 2014 CCS Guidelines [13]. Although the guidelines indicate that NOACs may be preferable over warfarin, we will group all anticoagulants together for the primary outcome because warfarin remains a suitable option for many patients. We acknowledge that anticoagulation may not be advisable in all these patients (e.g., high risk of bleeding, severe cognitive dysfunction making compliance unreliable, patient preference, etc.). However, it is impractical to accurately adjudicate appropriateness of withholding the anticoagulant using chart reviews alone given variability in charting practices and it is expected that the trial design will balance such patients across the study arms.

Secondary outcomes include the proportion of patients with the following characteristics: no CHADS2 risk factors and age <65 receiving anticoagulation; taking warfarin with at least six INRs measured per year; taking warfarin and time within a therapeutic range >60% using the Rosendaal method [73]; taking a NOAC with appropriate dosing (based on age and/or renal function); taking a NOAC but without renal function measured in 6 or 12 months; receiving other antiplatelets (e.g., aspirin alone, clopidogrel alone, aspirin plus dipyridamole, aspirin plus clopidogrel); taking an oral anticoagulant in combination with single or dual antiplatelet therapy; blood pressure (BP) and low-density lipoprotein (LDL) at target (to assess control of other stroke risk factors).

Process outcomes include the proportion of patients with AF in the cumulative patient profile, the proportion of patients with a formal stroke risk assessment completed, the proportion of patient charts with EMR-based tools used, and the proportion of providers who accessed the SAFIRE website to review their quality of care metrics.

### Measurement

EMR-based treatment data will be used to identify stroke prevention therapy. Previous work with EMRALD has shown that EMR data regarding active treatments (e.g., warfarin) is comparable to administrative data in terms of drug-capture for patients over age 65 (and far superior for patients under age 65 as drug administrative data in Ontario is limited in patients under 65) [16, 53]. To determine whether the stroke prevention therapy is concordant with guideline recommendations, the EMR data will be used to identify whether patients have relevant comorbidities (i.e., CHADS2 risk factors). The EMRALD team has validated, automated methods for identifying patients with diabetes [55, 74], ischemic heart disease [75], and hypertension [76] using data from both structured fields and free text. Patients identified with these conditions and relevant quality indicators are already included in the online feedback platform.

We developed a strategy for centralized identification of patients with AF in EMR data by performing a sensitive search for patients with a possible diagnosis of AF in the patient profile or with relevant treatments and/or results of cardiology tests that implicate a diagnosis of AF. An EMRALD team physician verified the charts to remove false positives from the study cohort, such as those with only brief runs of AF or those with only transient AF following surgery and follow-up testing that was normal. The methods for developing the search are similar to those used in previous studies [77] and based on previously described patient populations [16]. This search algorithm identified AF patients with one or more of the following components: (1) Recording of AF, including paroxysmal AF, “fibrillation”, and “flutter” in the problem list or history of past health fields of the EMR record [Additional file 3]; (2) electrocardiogram (ECG) and Holter monitor reporting AF; or (3) OAC prescription without clot, thrombosis, embolism, DVT, PE, valve replacement in the problem list, or history of past health fields, in combination with calcium channel blockers (CCB), ß-blockers, or digoxin prescription. The algorithm was able to accurately identify AF patients with a sensitivity of 78.6% (95% CI, 72.6–83.9%), a specificity of 99.9% (95% CI, 99.8–99.9%), positive predictive value of 95.1% (95% CI, 90.8–97.7%), and negative predictive value of 99.4% (95% CI, 99.1–99.5%). In addition, we developed and validated automated algorithms for identification of stroke and congestive heart failure to enable estimation of CHADS2 risk factors [78].

### Analysis

Outcomes will be compared at study completion at the patient, provider, and clinic levels. The analysis will be carried out on patient level variables using the generalized estimating equation approach to control for the effects of clustering. We will also adjust for the proportion of patients with guideline-concordant stroke prevention therapy at baseline and the other variables used in the minimization, as recommended by Taves [60]. Analysis will be performed on an intention-to-treat basis, and patient identifiers will be removed before transferring the data securely to analysts so that they will be blind to allocation. We will conduct the analysis as a longitudinal cohort design to permit exploration of patients whose treatments changed over time. As a sensitivity analysis, we will also conduct a repeated cross-sectional analysis to capture patients with newly developed AF during the intervention.

In keeping with similar trials in this literature, we considered a 10% difference in the primary outcome to be clinically important [48]. We assumed that 50% of patients with atrial fibrillation assigned to the usual care arm receive the primary outcome—using a control arm proportion of 50% is a conservative approach as it provides the largest sample size estimation. An EMRALD manual chart review of 7500 randomly selected adult patient charts discovered 192 patients with AF [16]; this 2.6% prevalence is similar to what has been reported in other primary care studies [79]. Based on that pilot data, we estimated that at least 100 patients would be available in each clinic (cluster). Therefore, using a reported intra-cluster correlation = 0.029 for warfarin uptake in patients with AF in primary care clinics [46], setting alpha = 0.05, and after adding 10% inflation for patient loss to follow up, 3276 patients (33 clinics) would be required to have 80% power [80].

### Recruitment

All eligible physicians in EMRALD have consented to participate in the study. Although this intervention is multifaceted, it is designed to limit the amount of non-clinical time required by the physicians. The intervention is meant to facilitate physicians to provide the right treatment to the right patient by leveraging available data to use at the point of care. The toolkit can be discussed with physicians at a time that suits them. The pragmatic approach does not require them to participate in ways they do not think will be valuable. As the investigators do not intervene directly upon patients, no patient-level consent process is required. Patients are not directly recruited but are analyzed if they remain part of the participating physician’s roster throughout the trial.

### Embedded process evaluation

We will be able to assess uptake of our toolkit by measuring the number of physicians and the frequency of log-ins to the SAFIRE website. We will also be able to measure the number of clinics that have uploaded our AF tools into their EMRs and the frequency of use and the number of physicians that actually used the tools in their clinical encounters. Finally, we will pursue a qualitative study to assess attitudes and barriers to uptake after the study is completed. Physician participants will be interviewed about the facilitators, barriers, or challenges to the uptake of the toolkit. The qualitative assessment and analysis will be informed by Normalization Process Theory which addresses how new practices are implemented, routinely embedded, and integrated in everyday practice through organizational, team, and individual perspectives [81].

## Discussion

### Strengths and limitations

The most important strength of this protocol is the use of a cluster-randomized trial design to test a clinically important question for large numbers of patients at high risk of stroke. The approach is pragmatic, highly valuing external validity by limiting eligibility criteria and testing a readily scalable suite of interventions tailored to address known barriers to optimal provision of stroke prevention therapy in primary care for patients with AF. We believe that the flexible toolkit approach will engender a sense that the intervention is partially “ground-up” (tailored and customized by participants) rather than completely “top-down” (implemented from the outside in a standardized fashion) leading to more meaningful use of the tools.

We acknowledge several potential limitations. First, the approach to delivering the interventions as a toolkit will make it difficult to determine which particular tools are most effective. While this approach will lead to variation in intervention uptake, it improves external validity, mimicking the real-world setting where providers will only adopt strategies perceived as useful in their context and for their patients. Second, although we have carefully developed tools to address known barriers, it is possible that other interventions may be valuable additions to the current approach or that the tools used will not be optimally tailored for the context. We have attempted to partially address this through a pre-trial phase including a mixed-methods pilot test in which the entire toolkit was tested in a primary care clinic, in keeping with the existing guidance for the development of complex interventions [82]. Third, our primary outcome is a process measure (i.e., appropriate prescribing) rather than a patient-oriented measure (e.g., stroke). We believe this is reasonable given the well-established effectiveness of stroke prevention therapy and believe this compromise is necessary given our desire to complete the analysis in a reasonable time frame. Fourth, our automated assessment of risk factors will be limited to data available in the EMR, and there is variation in the charting habits of family physicians. This may result in challenges for identification of patients with AF; patients with AF that cannot be readily identified will not be included in feedback reports and will not have the AF toolbar activated in their EMR chart. However, variation in charting is expected to be independent of randomization thus limiting risk of misclassification bias. Medications are entered as a structured variable in the chart and should not be affected by idiosyncratic data-entry. We recognize that some primary care clinics may be more likely to refer management of AF to consultants, but again expect that such characteristics would be independent of randomization. Further, if the rate of anticoagulation is far higher than expected, as seen in some studies [17], then we may encounter a ceiling effect. It is also possible that a ceiling effect may be reached due to external factors, such as attention given to this area by pharmaceutical companies. However, we may still find other results of interest in our analysis, including measures related to safety (i.e., appropriately frequent blood work). Finally, it is important to note that participants in EMRALD are a convenience sample of Ontario family physicians using PS Suite® EMR. This may make it difficult to generalize to other providers who use different EMR systems, or to those not using EMR at all. Nevertheless, most of the EMR strategies in the toolkit could be easily adapted to other systems.

### Implications

During the trial, the participants of EMRALD (both patients and providers) stand to benefit through improved tools and systems to manage this complicated problem in primary care. Increasing the proportion of patients with adequate stroke prevention therapy will result in improved outcomes for patients and for the health care system. If successful, similar multifaceted strategies may be developed and tested for other high-risk populations in this context.

## Additional files

Additional file 1: EMRALD Continuing Medical Education (CME) work plan work sheet. (DOCX 204 kb)
Additional file 2: Atrial fibrillation facts and reminders to use atrial fibrillation tools. (DOCX 279 kb)
Additional file 3: Terminology used to identify patients with atrial fibrillation within the electronic medical record. (DOCX 16 kb)

## Abbreviations

AF: Atrial fibrillation
BP: Blood pressure
CCB: Calcium channel blocker
CCS: Canadian Cardiovascular Society
CI: Confidence interval
CKD: Chronic kidney disease
CME: Continuing medical education
DVT: Deep vein thrombosis
ECG: Electrocardiogram
EMR: Electronic medical record
EMRALD: Electronic Medical Record Administrative data Linked Database
ICES: Institute for Clinical Evaluative Sciences
INR: International normalized ratio
IQR: Interquartile range
LDL: Low-density lipoprotein
NHS: National Health Service (UK)
NOAC: Novel oral anticoagulant
OAC: Oral anticoagulant
PE: Pulmonary embolism
QALY: Quality-adjusted life-years
SAFIRE: System for Audit and Feedback to Improve caRE
TIA: Transient ischemic attack
