# Supplementary material for: Improving stroke prevention therapy for patients with atrial fibrillation in primary care: protocol for a pragmatic, cluster-randomized trial
Source: Implement Sci. 2016 Dec 3;11:159. doi: 10.1186/s13012-016-0523-2 (PMC5135743; doi:10.1186/s13012-016-0523-2)
Supplement: Additional file 1: — EMRALD Continuing Medical Education (CME) work plan work sheet. (DOCX 204 kb) [file 13012_2016_523_MOESM1_ESM.docx]

# Additional file 1: EMRALD Continuing Medical Education (CME) work plan work sheet

#
