# Supplementary figures and images for: Improving stroke prevention therapy for patients with atrial fibrillation in primary care: protocol for a pragmatic, cluster-randomized trial
Source: Implement Sci. 2016 Dec 3;11:159. doi: 10.1186/s13012-016-0523-2 (PMC5135743; doi:10.1186/s13012-016-0523-2)

# Additional file 2: Atrial fibrillation facts and reminders to use atrial fibrillation tools


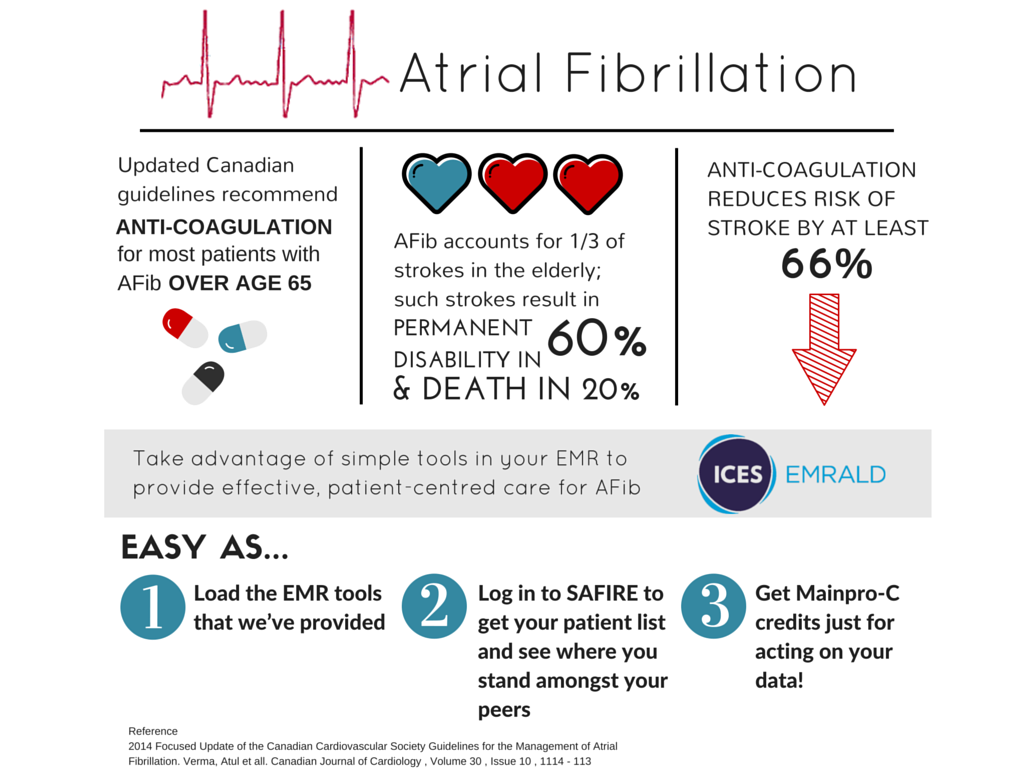

Supplement: Additional file 2: — Atrial fibrillation facts and reminders to use atrial fibrillation tools. (DOCX 279 kb) [file 13012_2016_523_MOESM2_ESM.docx]
