# Supplementary material for: Improving stroke prevention therapy for patients with atrial fibrillation in primary care: protocol for a pragmatic, cluster-randomized trial
Source: Implement Sci. 2016 Dec 3;11:159. doi: 10.1186/s13012-016-0523-2 (PMC5135743; doi:10.1186/s13012-016-0523-2)
Supplement: Additional file 3: — Terminology used to identify patients with atrial fibrillation within the electronic medical record. (DOCX 16 kb) [file 13012_2016_523_MOESM3_ESM.docx]

# Additional file 3: Terminology used to identify patients with atrial fibrillation with in electronic medical records

Atrial fibrillation

A-fibrillation

AF

Chronic atrial fibrillation

AFib

Paroxysmal atrial fibrillation (PAF)

Flutter

Fibrillation (not ventricular)
